# Myelin-specific IL2 + T-cells are associated with last occurring relapse severity in relapsing–remitting multiple sclerosis

**DOI:** 10.1038/s41598-026-39859-9

**Published:** 2026-02-14

**Authors:** Rina Zilkha-Falb, Tali Drori, Katya Shwartz, Michael Gurevich

**Affiliations:** 1 Multiple Sclerosis Center, Sheba Medical Center, Tel-Hashomer, Ramat Gan, Israel; 2 Department of Neurology, Gray Faculty of Medical and Health Sciences, Tel-Aviv University, Tel-Aviv, Israel

**Keywords:** Immunology, Neurology, Neuroscience

## Abstract

Autoreactive T-cells against myelin are implicated in multiple sclerosis (MS) however, the functional relevance of IL2-producing central memory T-cells and IFNɣ-producing effector T-cells have not been assessed. In this study we aimed to t investigate association of IL2 + and IFNγ + myelin reactive memory T-cells with MS severity and progression. Thirty relapsing–remitting MS (RRMS) patients and 32 age-matched healthy subjects (HS) were analyzed. Peripheral Blood Mononuclear Cells (PBMC) responses to proteolipid protein (PLP), myelin basic protein (MBP), and myelin oligodendrocyte glycoprotein (MOG) were assessed using IFNγ/IL2-FluoroSpot assay and correlated with disease severity and progression. Flow cytometry was performed to characterize central (CD45RO+CD62L+) and effector memory (CD45RO+CD62L-) T-cell subsets. Patients showed increased frequency and magnitude of IL2 responses to PLP compared with HS. Higher PLP-induced IL2 responses were in correlation with clinical severity of the last occurring relapse, including EDSS, EDSS change, and longer time from last occurring relapse (*p* < 0.05). Odds ratios (OR) confirmed associations between IL2-responses and last occurring relapse severity (OR range 5.3–22.5). Flow cytometry confirmed increase of central memory CD4 + and CD8 + T-cells in RRMS, whereas effector memory frequencies were unchanged. In RRMS, circulating PLP-reactive IL2 + central memory T-cells are linked to severity of last occurring relapse, representing an immunological imprint of recent disease activity rather than cumulative burden.

**Supplementary Information:**

The online version contains supplementary material available at 10.1038/s41598-026-39859-9.

## Introduction

Multiple sclerosis (MS) is a chronic immune-mediated disease of the central nervous system (CNS), characterized by inflammatory demyelination and progressive neurodegeneration. Breakdown of tolerance to myelin antigens, including myelin basic protein (MBP), proteolipid protein (PLP), and myelin oligodendrocyte glycoprotein (MOG), leads to the expansion of autoreactive T and B-cells and perpetuates CNS inflammation^1–5^. Genetic predisposition, environmental factors, and molecular mimicry have been implicated in the initiation of this process, culminating in microglial activation, pro-inflammatory cytokine release, and immune cell recruitment to the CNS^6,7^. Myelin-reactive T cells are present in both patients and healthy individuals, but differ in their functional profile and clinical relevance^3,8–11^.

Elevated IFNγ and IL10 responses to MBP and PLP have been demonstrated to correlate with disability in MS^10^, while MOG-specific IFNγ responses show weaker association with disease parameters^8^. These findings emphasize that both antigen specificity and functional phenotype of autoreactive T-cells determine their pathogenic significance.

Cytokine production further distinguishes effector from memory subsets. Effector T-cells typically produce IFNγ, driving acute inflammation, whereas central memory T-cells secrete IL2, enabling long-term persistence and proliferative renewal^12^. In MS, IFNγ-producing myelin-reactive T-cells expand during relapse^9^, but the role of IL2-producing myelin-reactive memory T-cells remains poorly defined. Previous studies have focused predominantly on Th1/Th17 cytokines (IFNγ, TNFα, GM-CSF, IL17, IL22)^8–10,13^, with limited attention to IL2/IFNγ secretion or to the memory phenotype of these autoreactive populations in relation to clinical outcomes. The present study focused on the simultaneous evaluation of IL2- and IFNγ-producing memory T-cells reactive to myelin antigens, characterization of their memory phenotype and the analysis of association with disease severity and progression. By integrating functional immune profiling with clinical parameters, this work aims to clarify the role of autoreactive memory T-cells in disease severity and progression.

## Materials and methods

### Study participants and design

Patients with clinically definite relapsing–remitting MS (RRMS), diagnosed according to the 2017 McDonald criteria^14^, were recruited at the Multiple Sclerosis Center, Sheba Medical Center, Ramat-Gan, Israel. Exclusion criteria for both RRMS patients and healthy subjects (HS) included a history of active autoimmune disease, chronic infection, or malignancy, as confirmed by medical records and self-reports. The study design is presented in Supplementary Fig. 1.

The study was approved by the Sheba Medical Center Institutional Review Board (approval no. SMC-7904-20). All research was performed in accordance with relevant guidelines/regulations and informed consent was obtained from all participants and/or their legal guardians.

Peripheral Blood Mononuclear Cells (PBMC) reactivity to MOG-, PLP-, and MBP-derived peptides was assessed using dual IFNγ/IL2 FluoroSpot assay. Memory T-cell phenotype was further characterized by flow cytometry in a subset of participants. Neurological evaluation, including Expanded Disability Status Scale (EDSS), was performed for all MS patients. Patients were routinely evaluated every 3–6 months and additionally during acute relapses. We have defined EDSS of last occurring relapse (EDSS score of the last occurring relapse before blood sampling), dEDSS (EDSS change during the last occurring relapse (dEDSS) was defined as the difference between the maximal EDSS documented during the last occurring relapse and the most recent EDSS recorded under remission prior to that relapse). Disease severity was assessed using the Multiple Sclerosis Severity Score (MSSS), calculated by standardizing EDSS according to disease duration. Annual Relapse Rate (ARR, total number of relapses standardized by disease duration) and correlation of these parameters with autoreactive PBMC responses was examined. Patients were free of corticosteroid treatment at the time of blood sampling. In accordance with common MS clinical-trial practice, a washout period of at least 2 months was considered sufficient to minimize corticosteroid-related immunological effects. One patient was sampled at relapse onset prior to corticosteroid administration.

### Cell preparation

PBMCs were isolated from EDTA-treated whole blood within 2 h of collection using Ficoll density gradient centrifugation. Cells were cryopreserved in medium containing 10% dimethyl sulfoxide (Sigma–Aldrich, Rehovot, Israel) and 90% cosmic calf serum (Hyclone, Utah, USA). Samples were frozen at − 1 °C/min and stored in liquid nitrogen (–196 °C) until analysis.

### FluoroSpot assay

Cryopreserved PBMCs were thawed in a 37 °C water bath and washed twice in culture medium (RPMI-1640 supplemented with 10% heat-inactivated cosmic calf serum, 2 mM L-glutamine, penicillin 100 U/ml, and streptomycin 100 µg/ml; Sigma-Aldrich). Viability was assessed using trypan blue staining (LUNA-II counter, Logos Biosystems), with a median viability of 88–90% and no significant group differences. For FluoroSpot, 300,000 viable PBMCs/well were seeded in pre-coated IFNγ/IL2 plates (Mabtech, FSP-0102) with anti-CD28 (0.1 µg/ml) and stimulated with overlapping peptide pools spanning MOG, PLP, or MBP, as well as appropriate controls. Magnitude of responses were quantified as spot-forming units (SFU). A response was considered positive if it exceeded the subject’s unstimulated background. Cumulative response to at least one of MOG, PLP, or MBP protein was defined as “any” protein.

To account for inter-individual variability, cytokine responses were normalized using protein-specific cut-off values defined as the upper 90% confidence interval of responses observed in healthy subjects. RRMS samples exceeding these thresholds were classified as myelin-reactive. For details see Supplementary Materials.

### Flow cytometry

Phenotypic analysis of central and effector memory subsets within CD4 + and CD8 + T- cells was performed using multiparameter flow cytometry (see Supplementary Materials). Frequencies of IL2– and IFNγ-producing cells were compared between MS patients and HS. Flow cytometry experiments were performed using pooled myelin peptides to assess the memory phenotype of myelin-reactive T cells; antigen specificity was determined exclusively by functional FluoroSpot assays using individual peptide pools.

### Statistical analysis

Continuous variables are reported as mean ± standard error (SE), and categorical variables as median with interquartile range (IQR, 25–75%). Between-group differences were tested with Student’s t-test or Mann–Whitney U-test, as appropriate. Fisher’s exact and Chi-square tests were used for categorical comparisons. Sensitivity analyses were performed to assess the robustness of cytokine response comparisons. Statistical outliers were identified using the 1.5× interquartile range (IQR) criterion and analyses were repeated after exclusion of these values. Results of sensitivity analyses were used to guide figure presentation and interpretation. Associations between autoreactive T-cell responses and clinical measures were assessed using Spearman’s correlation and odds ratios (OR). OR were calculated using binary logistic regression models, with corresponding 95% confidence intervals. For OR analyses, continuous clinical variables were dichotomized at the cohort median and analyzed using binary logistic regression. Statistical significance of ORs was assessed using the Wald test within the logistic regression framework. Statistical significance was defined as *p* < 0.05.

## Results

### Patient characteristics

The study included 30 patients with RRMS (mean age 42.9 ± 2.2 years; 24 females, 6 males) and 32 healthy subjects (HS; mean age 42.7 ± 2.8 years; 23 females, 9 males). In the RRMS group, the mean disease duration was 8.6 ± 1.7 years, the mean ARR was 0.5 ± 0.1, the median EDSS at blood sampling was 1.5 (IQR 1.0–2.0), the median time since of last occurring relapse was 0.5 years (0.2–1.9), and the median dEDSS was 1.0 (0.5–1.3) (Table 1).

**Table 1:** Demographic and clinical characteristics of participants.

Clinical characteristics	RRMS (*n* = 30),	HS (*n* = 32)	*p*-value
Age (years, mean ± SE)	42.9 ± 2.2	42.7 ± 2.8	0.87
Age of onset (years, mean ± SE)	35.7 ± 2.3	NA	
Gender (F/M)	24/6	23/9	0.4
Disease Duration in years (mean ± SE)	8.6 ± 1.7	NA	
EDSS at blood sampling [median (IQR)]	1.5 (1.0–2.0)	NA	
Annual Relapse Rate (mean ± SE)	0.50 ± 0.08	NA	
Time from last occurring relapse [years, median (IQR)]	0.50 (0.20–1.85)	NA	

All patients were steroid-free at the time of blood sampling. One patient was sampled at relapse onset prior to corticosteroid administration. F - female; M - male; NA - not applicable; RRMS - relapsing–remitting multiple sclerosis; HS - Healthy Subjects; IQR - Interquartile Range; SE – standard error.

### Magnitude of IL2 and IFNγ secretion by PBMC in response to myelin proteins

**Fig. 1:**
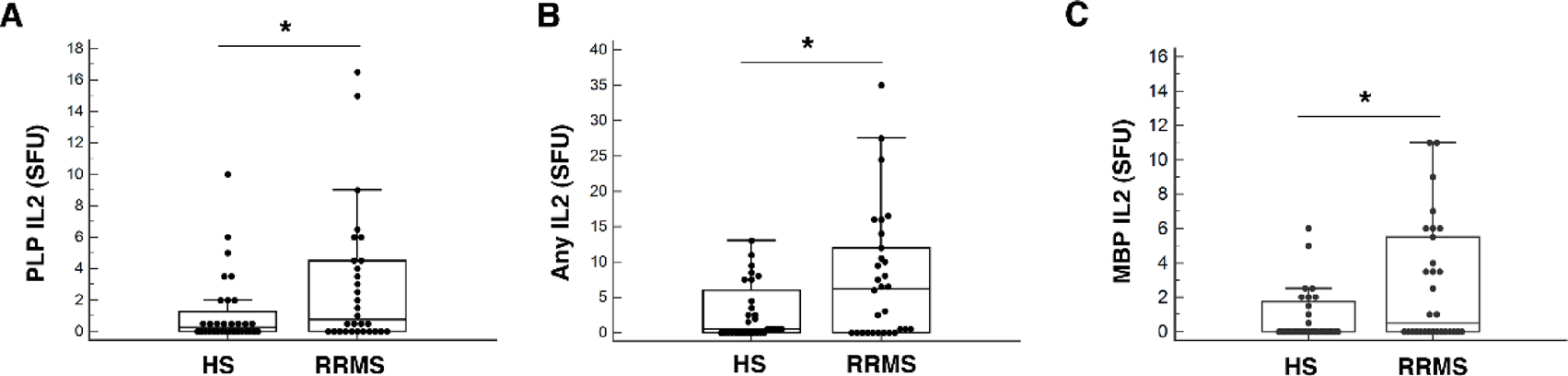
Magnitude of IL2 response to myelin proteins in RRMS as compared with HS. Median (SFU) of IL2 production by PBMC in response to PLP (**A**), any protein (**B**) and MBP (**C**). *p < 0.05. Sensitivity analyses excluding statistical outliers (1.5× IQR) were performed; IL2–related results remained significant, whereas myelin-induced IFNγ responses were not and are therefore not shown. Horizontal bars represent medians; boxes indicate interquartile range.

In FluoroSpot analysis PBMC from RRMS patients exhibited significantly higher IL2 secretion in response to PLP [0.8 (0.0–4.5) vs. 0.3 (0.0–1.6) SFU, *p* < 0.05] and to any protein [6.3 (0.0–12.5) vs. 0.5 (0.0–2.5) SFU, p=0.04] compared with HS. A higher response was also observed for MBP [0.5 (0.0–5.6) vs. 0.0 (0.0–1.5) SFU, *p* < 0.05] (Fig. 1A-C; Table 2). In contrast, the difference observed in response to PLP MOG, MBP, and any protein by IFNγ secretion was no longer statistically significant. Notably, cytokine responses to non-specific stimulation (anti-CD3/CD28) were comparable between RRMS and HS (See Supplementary Materials), supporting the antigen specificity of myelin responses.

**Table 2:** Magnitude of IL2 and IFNγ secretion by PBMC in responses to Myelin proteins.

Characteristics	IL-2				IFNg			
Myelin proteins	MOG	PLP	MBP	Any	MOG	PLP	MBP	Any
PBMC response in HS [SFU, median (IQR)]	0.0 (0.0-0.5)	0.30 (0.0-1.6)	0.0 (0.0-1.8)	0.50 (0.0-2.5)	1.50 (0.0-3.4)	0.0 (0.0-1.5)	0.0 (0.0-1.3)	3.00 (0.13–4.5)
PBMC response in MS [SFU, median (IQR)]	0.25 (0.0-2.6)	0.75 (0.0-4.5)	0.50 (0.0-5.6)	6.25 (0.0-12.5)	1.75 (0.5–5.2)	1.00 (0.0–4.00)	0.0 (0.0-1.1)	4.25 (0.75–11.1)
p-value	NS	< 0.05	<0.05	0.04	NS	NS	NS	NS
Cut offs for positive PBMC response in MS (SFU)*	> 4.5	> 3.5	> 2.5	> 9.5	> 6.5	> 2.5	> 3.0	> 13.0

*Shown also cut off levels for magnitude of IL2 and IFNγ secreting PBMC in response to myelin proteins in RRMS patients calculated as upper level of 90% confidence intervals in HS. Any - refers to response to at least one protein of either MOG, PLP or MBP; NS - not significant; SFU – Spot Forming Unit.

### Normalized frequencies of myelin-reactive PBMC

**Fig. 2:**
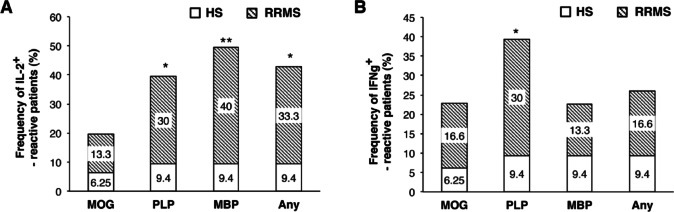
Normalized frequencies of IL2 and IFNγ-producing PBMC in response to myelin proteins in RRMS patients. Frequencies of subjects producing IL2 (**A**) and IFNγ (**B**) by PBMC in response to MOG, PLP, MBP or any protein in RRMS as compared with HS. Subsequent analyses of cytokine magnitude were restricted to myelin-reactive individuals, defined by exceeding healthy subject–derived cut-off values. *p < 0.05, **p < 0.005.

Given heterogeneity among HS, the upper level of 90% confidence interval (CI) of HS responses was used as a cut-off for defining positive responses in RRMS. Cut-offs were > 4.5, > 3.5, > 2.5, and > 9.5 SFU for IL2 responses to MOG, PLP, MBP, and any protein, respectively; and > 6.5, > 2.5, > 3.0, and > 13.0 SFU for IFNγ (Table 2). Based on these thresholds, RRMS patients displayed significantly higher frequencies of IL2-reactive PBMC to PLP (30.0% vs. 9.4%, *p* = 0.04), MBP (40.0% vs. 9.4%, *p* = 0.005), and any protein (33.3% vs. 9.4%, *p* = 0.02) compared with HS (Fig. 2A, B). For IFNγ-reactive PBMC frequencies were higher for PLP (30.0% vs. 9.4%, *p* = 0.04), while differences for other proteins were not significant. Although low-level heterogeneity of myelin-reactive responses was observed in healthy subjects, elevated responses exceeding the HS-derived cut-off values were detected predominantly in RRMS patients (13–40%), compared with < 10% of healthy subjects.

Among RRMS myelin-reactive patients, median IL2 secretion reached 10.0 SFU for MOG, 6.3 for PLP, 6.0 for MBP, and 16.0 for any protein. Median IFNγ responses were 10.0, 7.5, 4.5, and 41.8 SFU, respectively. In contrast, median values in HS were zero across all conditions (*p* < 0.0001).

### Association of IL2 responses to Myelin proteins with clinical parameters

Comparison of clinical variables between myelin-reactive and non-myelin-reactive patients revealed that IL2 responses to PLP and to any myelin protein have more severe last occurring relapse. Specifically, PLP-reactive patients showed significantly higher dEDSS increase at last occurring relapse (2.3 ± 0.6 vs. 0.8 ± 0.1; *p* = 0.02), and tendency to longer time since last occurring relapse 4.3 ± 2.0 vs. 1.7 ± 0.8 years; *p* = 0.07) (Table 3).

**Table 3:** Comparison of RRMS clinical parameters in myelin-reactive by secreting IL2 and non-myelin-reactive patients.

Clinical parameters	Group	MOG	PLP	MBP	Any
dEDSS	Myelin-reactive	3.1 ± 1.4	2.3 ± 0.6	1.4 ± 0.4	2.5 ± 1.9
	Non-myelin-reactive	1.1 ± 0.1	0.8 ± 0.1*	1.2 ± 0.3	0.8 ± 0.6*
EDSS last occurring relapse	Myelin-reactive	5.3 (3.2–6.0)	2.0 (2.0–5.0)	2.0 (2.0-3.1)	2.0(0.4–2.8)
	Non-myelin-reactive	2.0 (0.5–9.6)**	2.0(2.0-2.7)	2.0(2.0–3.0)	2.0(2.0-2.9)
Disease duration (years)	Myelin-reactive	7.8 ± 2.8	10.8 ± 2.2	8.3 ± 1.9	9.0 ± 1.9
	Non-myelin-reactive	8.7 ± 1.9	7.6 ± 2.2	9.1 ± 2.6	8.3 ± 2.4
Time from last occurring relapse (years)	Myelin-reactive	1.1 ± 0.5	4.3 ± 2.0	3.4 ± 1.5	2.8 ± 1.3
	Non-myelin-reactive	2.7 ± 0.9	1.7 ± 0.8 (0.1)	1.9 ± 1.2*	2.4 ± 1.4
MSSS	Myelin-reactive	0.3 ± 0.1	0.3 ± 0.1	0.5 ± 0.2	0.4 ± 0.1
	Non-myelin-reactive	0.5 ± 0.2	0.3 ± 0.1	0.3 ± 0.1	0.3 ± 0.1
ARR	Myelin-reactive	0.6 ± 0.2	0.3 ± 0.1	0.6 ± 0.1	0.4 ± 0.1
	Non-myelin-reactive	0.5 ± 0.1	0.6 ± 0.1	0.5 ± 0.1	0.4 ± 0.1

*Any, refers to response to at least one protein of either MOG, PLP or MBP. * *p* < 0.05; ** *p* < 0.02; *** *p* < 0.01.

**Fig. 3:**
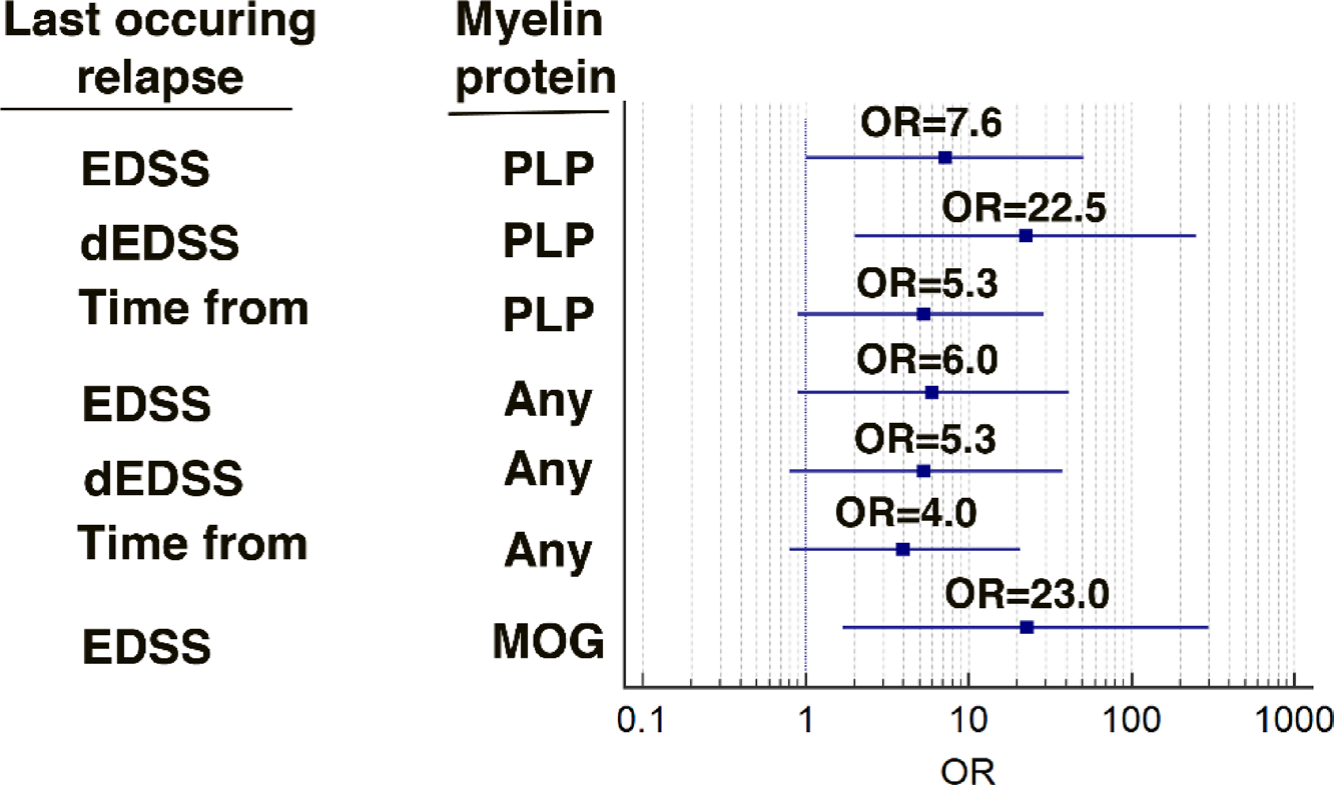
Strength of association between clinical parameters of last occurring relapse and responses to myelin proteins by IL2 production.Effect of clinical parameters of last occurring relapse on frequency of PLP any myelin protein and MOG-reactive patients demonstrated by OR. Odds ratios were calculated using dichotomized clinical variables; identical median values do not preclude differences in distribution across categories. EDSS, dEDSS and “Time from” are referred to last occurring acute relapse.

Odds ratio analysis confirmed strong associations between higher IL2 responses to PLP and higher last occurring relapse severity: EDSS at last occurring relapse (OR 7.6, 95% CI 1.0–51.4, *p* = 0.02), dEDSS of last occurring relapse (OR 22.5, 95% CI 2.0–249.2, *p* = 0.005), and time since last occurring relapse (OR 5.3, 95% CI 0.96–29.3, *p* = 0.03) (Fig. 3). Noteworthy, the tendency of longer time from last occurring relapse in PLP-reactive patients was confirmed by significant OR.

Any protein-reactive patients also showed greater dEDSS (2.5 ± 1.9 vs. 0.8 ± 0.6; *p* = 0.02), while OR revealed associations with higher EDSS of last occurring relapse (OR 6.0, 95% CI 0.9–41.4, *p* = 0.03), dEDSS of last occurring relapse (OR 5.3, 95% CI 0.8–37.1, *p* = 0.04), and time since last occurring relapse (OR 4.0, 95% CI 0.8–20.9, *p* < 0.05) (Fig. 3). MOG-reactive patients showed significant difference only in EDSS at last occurring relapse (5.3 (3.2-6.0) vs. 2.0 (0.5–9.6) *p* = 0.03), with significant association by OR analysis (OR 23.0, 95% CI 1.7–298.4; *p* = 0.008).

MBP-reactive patients are characterized by longer time from last occurring relapse (3.4 ± 1.5 vs. 1.9 ± 1.2 years, *p* < 0.05), however these results were not confirmed by OR analysis.

### Association of IFNγ responses to Myelin proteins with clinical parameters

In contrast to IL2, PLP- and any protein-reactive patients by IFNγ secretion are characterized by shorter time from last occurring relapse (0.8 ± 0.3 vs. 3.2 ± 1.2 years and 0.6 ± 0.1 vs. 2.9 ± 2.2, *p* < 0.05). In addition, PLP-reactive patients have higher annual severity (0.4 ± 0.2 Vs 0.1 ± 0.1, *p* < 0.05) (Table 4), but these associations were not confirmed by OR analysis.

**Table 4:** Comparison of clinical parameters in IFNγ myelin-reactive and non-myelin-reactive RRMS patients.

Clinical parameters		MOG	PLP	MBP	Any
dEDSS	Myelin-reactive	2.2 ± 0.9	1.7 ± 0.6	1.1 ± 0.3	2.2 ± 0.9
	Non-myelin-reactive	1.1 ± 0.2	1.1 ± 0.3	1.6 ± 0.3	1.1 ± 0.2
EDSS last occurring relapse	Myelin-reactive	2.5(2.0–5.0)	2.0(2.0–3.0)	2.0(2.0-2.7)	2.5(2.0–5.0)
	Non-myelin-reactive	2.0(2.0–3.0)	2.0(2.0–3.0)	2.0(2.0–3.0)	2.0(2.0–3.0)
Disease duration (years)	Myelin-reactive	7.4 ± 3.7	7.9 ± 1.8	11.8 ± 4.0	7.4 ± 3.7
	Non-myelin-reactive	8.8 ± 1.9	9.4 ± 2.7	8.0 ± 1.8	8.8 ± 1.9
Time from last occurring relapse (years)	Myelin-reactive	5.6 ± 3.4	0.8 ± 0.3	5.6 ± 3.4	0.6 ± 0.1
	Non-myelin-reactive	2.0 ± 0.8	3.2 ± 1.2*	2.0 ± 0.8	2.9 ± 2.2*
Annual severity	Myelin-reactive	0.5 ± 0.4	0.4 ± 0.2	0.2 ± 0.1	0.5 ± 0.3
	Non-myelin-reactive	0.4 ± 0.1	0.1 ± 0.1*	0.3 ± 0.1	0.2 ± 0.1
ARR	Myelin-reactive	0.6 ± 0.2	0.5 ± 0.2	0.5 ± 0.2	0.6 ± 0.2
	Non-myelin-reactive	0.5 ± 0.1	0.5 ± 0.1	0.5 ± 0.15	0.5 ± 0.1

*Any refers to response to at least one protein of either MOG, PLP or MBP. * *p* < 0.05.

### Memory CD4 + and CD8 + T-cell phenotypes in RRMS and HS

To further characterize the phenotype of myelin-reactive T-cells, PBMCs from six RRMS patients (mean age 38.9 ± 3.3 years; 5 females/1 male; median EDSS 2.0 [0.5–2.75]; mean disease duration 10.4 ± 3.2 years) and five age- and sex-matched HS (mean age 38.3 ± 6.5 years; 3 females/2 males) were analyzed by flow cytometry.

**Fig. 4:**
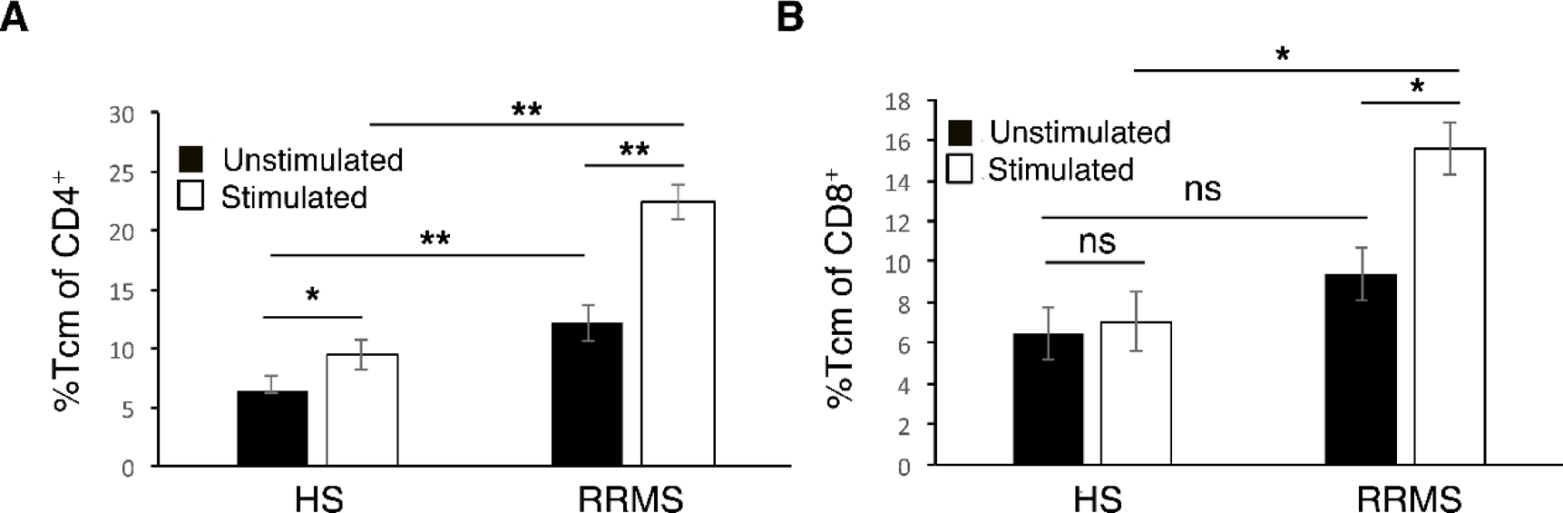
Frequencies of central memory T-cells phenotypes in response to myelin proteins in RRMS as compared with HS. Central memory T-cells were characterized by CD45RO+/CD62L+ in CD4 (**A**) and CD8 (**B**) lymphocytes. *p < 0.05, **p < 0.001.

In the RRMS group, stimulation with pooled MOG, PLP, and MBP peptides induced a two-fold increase in proportion of central memory CD4 + T-cells (CD4+/CD45RO+/CD62L+) compared with unstimulated cells (24.1 ± 3.9% vs. 12.2 ± 1.3%, *p* = 0.005). A similar, though less pronounced, effect was observed in HS, where central memory CD4 + T cells increased 1.5-fold upon stimulation (9.4 ± 1.2% vs. 6.4 ± 0.2%, *p* = 0.03). Importantly, the frequency of central memory CD4 + T-cells was significantly higher in RRMS compared with HS, both in unstimulated (*p* = 0.001) and myelin-stimulated conditions (*p* = 0.009) (Fig. 4A).

For CD8+ T-cells, RRMS patients also demonstrated higher central memory frequencies upon myelin stimulation, showing a 1.7-fold increase (16.4 ± 1.5% vs. 9.4 ± 1.6%, *p* = 0.04). In contrast, HS did not exhibit a significant change after stimulation (7.1 ± 2.1% vs. 6.4 ± 1.6%, *p* = 0.4). While baseline frequencies of central memory CD8 + T-cells were not significantly different between RRMS and HS (*p* = 0.1), myelin-stimulated frequencies were significantly higher in RRMS (*p* = 0.02) (Fig. 4B).

In contrast to these findings, effector memory T-cell (CD4+/CD45RO+/CD62L-) frequencies did not differ significantly between RRMS and HS, regardless of stimulation (See Supplementary Materials).

Finally, we performed a post hoc statistical comparison of sex distribution between the RRMS (5 females/1 male) and healthy control (3 females/2 males) groups using a Chi-square test. This analysis revealed no statistically significant difference in sex distribution between groups (*p* > 0.05), indicating that the slight imbalance is unlikely to confound the observed differences in memory T-cell phenotypes.

## Discussion

In this study, we provide a functional characterization of IL2 – and IFNγ-myelin reactive PBMC in response to major myelin antigens (PLP, MOG, MBP) in RRMS patients and their association with clinical measures of disease severity. We found that PBMC from RRMS patients exhibited significantly increased IL2 responses to PLP compared with HS, and that IL2 production,particularly in response to PLP and to any myelin protein,was associated with clinical measures of last occurring relapse severity including EDSS and dEDSS of last occurring relapse and time since the last occurring relapse. These functional findings were paralleled by flow cytometric evidence of increased central memory (CD45RO+CD62L+) CD4+ and CD8+ T-cell frequencies in RRMS, supporting the expansion of autoreactive central memory populations. The increased IL2 secretion was observed in parallel with an increased frequency of central memory T-cells, suggesting a possible association between IL2–producing myelin-reactive responses and the central memory compartment. These findings suggest that in periphery autoreactive memory expansion in RRMS is preferentially driven by central memory rather than effector memory subsets. Importantly, the auto-reactive T-cells were associated with last occurring acute relapse-related parameters rather than cumulative disease measures, suggesting that myelin-reactive IL2+ central memory cells may primarily reflect immunological imprints of recent disease activity.

To our knowledge, this is the first study to directly link the frequency and magnitude of PLP-reactive IL2+ central memory T-cells with clinical severity of last occurring relapses in RRMS. These data extend earlier reports of autoreactive T-cell responses in MS by demonstrating not only their presence but also their functional association with short-term disease outcomes. Prior studies have described autoreactive responses to myelin antigens across different cytokine profiles, including IL10, IL17, IL22, IL23, and IFNγ^8,10,13^. Consistent with our findings, Moldovan et al. (2003) reported increased PLP-induced IL10 responses in MS, whereas MBP-induced IFNγ responses were elevated only in mildly disabled patients^10^. The divergence from our results may be explained by clinical differences, as our cohort included patients with more advanced disability. Similarly, Tejada-Simon et al. (2001) reported a lack of IFNγ production by PLP-reactive T cells, whereas MBP responses were more prominent,differences that may reflect variability in peptide pools and patient cohorts. In contrast, Bronge et al., (2019) demonstrated elevated MOG-specific IFNγ responses using a bead-based stimulation method^8^, while we observed weaker MOG reactivity, aligning instead with earlier ELISpot and proliferation studies that failed to detect consistent MOG responses^3,15,16^. These methodological differences highlight how antigen presentation strategies and patient conditions critically shape the observed autoreactivities.

Our choice of IL2 and IFNγ as readouts was based on functional distinctions between central memory IL2 + and effector memory IFNγ + T Cells subsets^12^. Indeed, the correlation of frequency of PLP-induced IL2 reactive patients with last occurring relapse severity supports the role of autoreactive central memory cells as persistent drivers of disease, whereas IFNγ responses were less consistently associated with clinical and immunophenotypic parameters compared with IL2 responses. This interpretation is consistent with Lunemann et al. (2008), who described EBNA1-specific CD4 + T cells cross-reactive with myelin antigens, co-producing IL2 and IFNγ, and bearing central/effector memory phenotypes^17^. In this context, interesting is the observation of positive correlation of time from last occurring relapse with central memory in myelin-reactive patients (IL2+), while negative associations observed for the effector memory T-cells myelin-reactive patients (IFNγ+). This observation is consistent with the general concept that IL2–producing central memory T-cells are associated with sustained immune memory, whereas IFNγ-producing effector T-cells reflect more immediate, context-dependent effector activity^12,17,22^.

A striking feature of our study was the antigen specificity of responses. While HS and RRMS showed similar cytokine secretion after non-specific CD3/CD28 stimulation, significant IL2 responses were observed only to myelin peptides in RRMS. Among the myelin proteins, PLP elicited the strongest and most consistent IL2 responses, in line with prior reports identifying PLP as a dominant autoantigen in subsets of MS patients^18–20^. The surface-exposed MOG and PLP that spans the myelin sheath multiple times, potentially creating unique immunogenic epitopes^21^. This could explain why PLP- and MOG-reactive IL2 + responses were linked to more severe last occurring relapses, compared with the more internally localized MBP.

Our phenotypic analysis further demonstrated that central memory CD4 + and CD8 + T-cells were significantly expanded upon myelin stimulation in RRMS but not in HS. In contrast, effector memory populations did not differ. These findings reinforce the interpretation that autoreactive central memory rather than effector memory subsets underlying the associations with last occurring relapse severity. Similar results were described by Okuda et al. (2005), who found elevated total memory CD4+ populations during exacerbations and persistently increased CD8+ memory cells in RRMS^22^.

Interestingly, the correlation of central memory T-cells with clinical presentation of last occurring relapse suggesting that circulating IL2+ memory T-cells may be transiently mobilized during or after relapses and supports the concept that autoreactive memory T-cells in circulation may reflect the most recent immunological events rather than cumulative history. Since they are long-lived, they can gradually increase in number between relapses as part of the natural maintenance of memory T-cell pools. This re-expansion could explain the positive correlation with the time from last occurring relapse. This correlation corroborated with previous study that analyzed general population of memory T-cells^23^.

This study has several limitations. First, the cross-sectional design prevents causal inference, and longitudinal studies are required to validate the results. Second, the relatively small cohort limits generalizability, and replication in larger, more diverse populations is warranted. Given the limited number of myelin-reactive patients, analyses of clinical associations and odds ratios should be considered exploratory. These findings require confirmation in larger cohorts and should be interpreted with caution. An additional limitation of this study is that cytokine secretion and memory T-cell phenotype were assessed using two separate experimental approaches. Cytokine production was measured in PBMC cultures by FluoroSpot, while memory T-cell subsets were evaluated by flow cytometry without intracellular cytokine staining, thus, integration of these results reflects an inferred association rather than direct single-cell attribution. Furthermore, the relatively low frequency of myelin-induced cytokine responses limits statistical power and supports interpretation of the findings as exploratory. Finally, flow cytometry was performed using pooled peptide mixtures, limiting resolution of antigen-specific clonotypes.

Overall, our data highlight the functional role of circulating PLP-reactive IL2+ central memory T-cells in RRMS. The frequency of these responses is associated with last occurring relapse severity and reflect acute disease activity rather than cumulative burden. Further studies should determine whether these responses can serve as biomarkers for disease monitoring and therapeutic stratification.

## Supplementary Information

Below is the link to the electronic supplementary material.

Supplementary Material 1

## Data Availability

Raw data available from corresponding author upon request.
