# Supplementary material for: Myelin-specific IL2 + T-cells are associated with last occurring relapse severity in relapsing–remitting multiple sclerosis
Source: Sci Rep. 2026 Feb 14;16:9011. doi: 10.1038/s41598-026-39859-9 (PMC12992694; doi:10.1038/s41598-026-39859-9)
Supplement: Supplementary file 1 — Supplementary Material 1 [file 41598_2026_39859_MOESM1_ESM.pdf]

Myelin-Specific IL2+ T-Cells Are Associated with Last Occurring Relapse Severity in Relapsing–Remitting Multiple Sclerosis

Rina Zilkha-Falb, Tali Drori, Katya Shwartz and Michael Gurevich

Supplementary Materials.

Supplementary Figure 1. Study design.

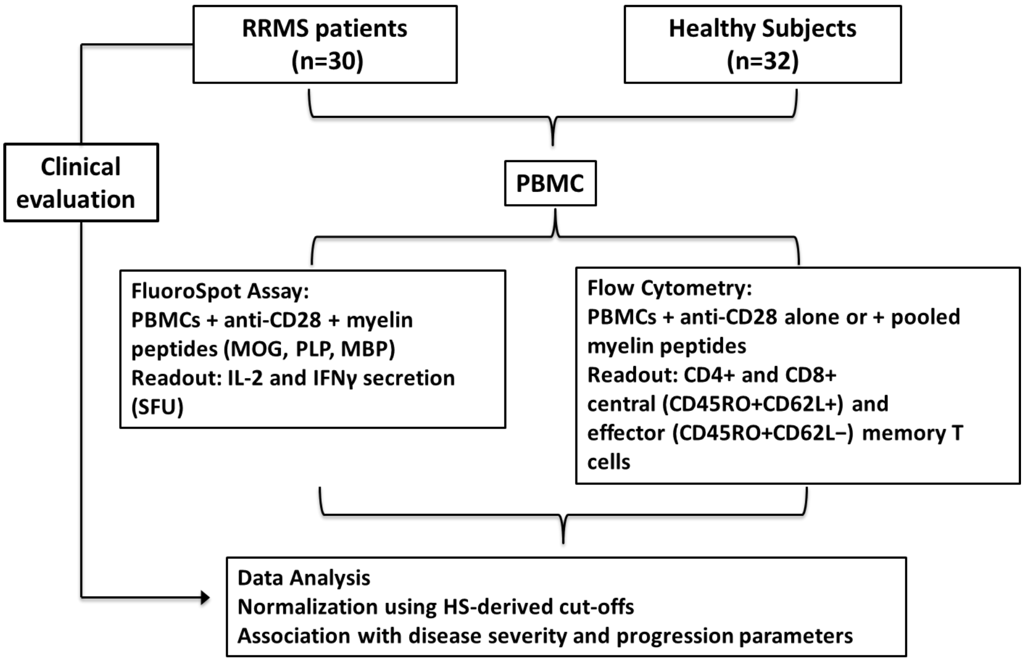

FluoroSpot assay

Cryopreserved PBMCs were briefly thawed in a water bath at 37°C before washing twice in culture media (CM) [RPMI 1640 medium (R8758, Sigma-Aldrich, Rehovot, Israel) supplemented with 10% heat-inactivated cosmic calf serum (Hyclone Laboratories Utah, USA), 2 mM l-glutamine (G7513, Sigma-Aldrich), and penicillin (100 U/ml) and streptomycin (100 µg/ml) (P4333, Sigma-Aldrich)]. Cell count and viability were measured by trypan blue staining using an automated counter (LUNA-II, Logos Biosystems).

A total of 300,000 viable PBMC in 100 µl of CM per well were added to a pre-coated and blocked IFNγ/IL-2 FluoroSpot plate (FSP-0102, Mabtech) containing 100 µl of

CM and anti-CD28 (0.1 μg/ml) according to manufacturer protocol and co-stimulated with overlapping peptides pool of either myelin protein MOG, PLP and MBP as compared with appropriate controls.

PBMC were co-stimulated with overlapping peptides pool of either myelin protein MOG, PLP and MBP scanning peptides pool provided each as a pool represents the whole human protein [PepTivatorMOG cat. 130-096-770, PLP cat. 130-097-274 and MBP cat. 130-097-287 (Miltenyi Biotec), respectively; each at 1 μg/ml final concentration]. The peptides are 15-mers overlapping with 11 amino acids, covering the sequence of each protein.

For the non-specific (positive control) anti-CD3 stimuli (0.1 μg/ml), 50,000 PBMCs were added to each well. The plates were incubated overnight in a humidified incubator (5% CO_2, 37°C). Each test was performed in duplicates, and MS samples were tested in parallel with control (from HS) samples on the same plate. After incubation, the plates were developed according to the manufacturer's instructions. The FluoroSpot plates were read using an IRIS plate reader (Mabtech, Sweden) and the IRIS software version 1.1.9 (Mabtech AB). For each tested subject, the spontaneous IL2- and IFNγ-production by the PBMCs in unstimulated wells (in CM and anti-CD28 only as background) were subtracted from the myelin-induced -IL2 and -IFNγ response, respectively. A response was considered positive if the reactivity to an overlapping peptide pool of either MOG, PLP or MBP exceeded the cut-off value (individual subject's background).

For MS patients, the positive cutoff value was set above the 90% confidence interval of specific myelin protein response observed in HS.

Indeed, the sum of responses for the three myelin-protein-derived overlapping peptides was considered, which address the response for at least one protein responsiveness and defined as any protein.

Spot forming units was referred as SFU; any protein - referred to magnitude (SFU) or frequencies of response for least one from MOG, PLP or MBP myelin protein

Flow cytometry

Phenotypic characterization and frequency analysis of central and effector memory T cells within the total population of CD4+/CD8+ T cells were performed using the FACS method. For the phenotype characterization PBMCs aliquots were thawed in a water bath washed using CM and rested overnight in a humidified incubator (5% CO_2, 37 °C). Then cells were stimulated with a mixture of MOG, PLP and MBP (each at 1 μg/ml final concentration) in presence of anti-CD28 (0.1 μg/ml) for 24 hours. Parallel cells cultured in presence of anti-CD28 and absence of myelin proteins mixture served as control. After incubation the cells were collected into FACS tubes, washed with FACS buffer (PBS 1%, BSA 10%, NaN3 0.1%), and antibodies with the following specific membrane markers were added: (CD4, CD8, CD45RA, CD45RO, CD62L). The cells were fixed using 1% paraformaldehyde and subjected to analysis in the Gallios flow cytometer (Beckman Coulter). PBMCs were characterized in the absence of myelin peptides (anti-CD28 alone) and after stimulation with myelin peptide mixture (MOG, PLP, MBP) in the presence of anti-CD28. Unstimulated cells represent the total frequency of memory T cells, while stimulated cells represent the frequency of myelin-specific memory T cells.

Comparable magnitude of cytokine secretions between HS and RRMS in response to general stimulation

Spontaneous IFNγ and IL2 production by PBMC was measured by culturing cells in the absence of antigen. In parallel, we examined the response to stimulation with anti-CD28 and anti-CD3 as general stimulation.

To ensure that the responses to MOG, PLP and MBP were not due to an over-representation of cytokine producing T-cells in general, a comparison between the responses to the polyclonal T-cell activator anti-CD3 Ab was performed.

There was a large inter-individual variation, but no significant difference of responses to CD28 and CD3 between patients in the RRMS and HC groups was observed for either IL2 or IFNγ. The frequency of RRMS myelin-reactive patients and HC was 93% and 96% for IL2 and 100% for IFNγ, respectively. The mean SFU for IL2 and IFNγ was 63.8±14.9 vs 69.2±14.2 p=0.5 and 388.1±76.7. vs 308.0±51.2 p=0.3 in MS and HS, respectively.

Analysis of effector memory CD4+ and CD8+ T Cells phenotype

The frequency of effector memory (Tem) $CD4^+$ T cells ($CD4^+CD45RO^+CD62L^-$) in PBMCs did not differ significantly between myelin-stimulated and unstimulated conditions in either RRMS patients or healthy subjects. Likewise, no significant differences were observed between RRMS and healthy subjects under either condition.

Similarly, the frequency of effector memory $CD8^+$ T cells ($CD8^+CD45RO^+CD62L^-$) was comparable between myelin-stimulated and unstimulated PBMCs in both RRMS patients and healthy subjects, with no significant differences between groups.

Supplementary Table 1.

Effector memory CD4+ and CD8+ T Cells upon myelin stimulation.

**Cell subset**	**Group**	**Unstimulated (%) Mean ± SE**	**Myelin-stimulated (%) Mean ± SE**	**p value**
$CD4^+$ Tem ($CD45RO^+CD62L^+$)	RRMS	14.1±4.1	14.4±3. 4	0.47
	HS	23.3±9.7	21.1±11.4	0.44
$CD8^+$ Tem ($CD45RO^+CD62L^+$)	RRMS	29.8±5.4	28.2±5.4	0.47
	HS	23.6±5.1	21.1±4.7	0.3

Tem – effector memory T-cells
